# Task-based memory systems in contextual-cueing of visual search and explicit recognition

**DOI:** 10.1038/s41598-020-71632-4

**Published:** 2020-10-05

**Authors:** Thomas Geyer, Pardis Rostami, Lisa Sogerer, Bernhard Schlagbauer, Hermann J. Müller

**Affiliations:** 1 Department Psychologie, Lehrstuhl für Allgemeine Und Experimentelle Psychologie, Ludwig-Maximilians-Universität München, Leopoldstraße 13, 80802 München, Germany; 2 Munich Center for Neurosciences – Brain and Mind, Ludwig-Maximilians-Universität München, Planegg-Martinsried, Germany

**Keywords:** Neuroscience, Psychology

## Abstract

Visual search is facilitated when observers encounter targets in repeated display arrangements. This ‘contextual-cueing’ (CC) effect is attributed to incidental learning of spatial distractor-target relations. Prior work has typically used only one recognition measure (administered after the search task) to establish whether CC is based on implicit or explicit memory of repeated displays, with the outcome depending on the diagnostic accuracy of the test. The present study compared two explicit memory tests to tackle this issue: yes/no recognition of a given search display as repeated versus generation of the quadrant in which the target (which was replaced by a distractor) had been located during the search task, thus closely matching the processes involved in performing the search. While repeated displays elicited a CC effect in the search task, both tests revealed above-chance knowledge of repeated displays, though explicit-memory accuracy and its correlation with contextual facilitation in the search task were more pronounced for the generation task. These findings argue in favor of a one-system, explicit-memory account of CC. Further, they demonstrate the superiority of the generation task for revealing the explicitness of CC, likely because both the search and the memory task involve overlapping processes (in line with ‘transfer-appropriate processing’).

## Introduction

Humans display an impressive capability for extracting statistical regularities from encountered scenes^1^. Thus, for example, finding a searched-for ‘target’ item, such as some product in the supermarket, is facilitated by having found it repeatedly at the same location within a predictable arrangement relative to other items. In a comparable laboratory setting, observers are likewise faster in detecting a target letter embedded in a set of non-target, or distractor, letters when the spatial arrangement of the search items is repeated across trials. Such repeatedly encountered target-distractor arrangements, or ‘contexts’, come to guide visual search, ‘cueing’ attention to the target location (i.e., contextual- cueing effect)^2^. While contextual cueing is a well-established facilitator in visual search tasks^3^, there is an ongoing controversy whether cueing is reliant on a unitary—explicit-memory system^4,5^, or whether it is more appropriate to assume two independent—implicit and explicit-memory systems supporting contextual cueing of search and recognition of encountered scenes, respectively^6,7^. The current experiment used a novel factorial approach^8^ of employing qualitatively different awareness tests to investigate their ability to reveal explicit contextual cueing and to test which memory model is most likely to explain the observed effect patterns.

### Explicit memory in contextual cueing

In their seminal study, Chun and Jiang^2^ proposed that contextual cueing is an implicit effect—though this has become a controversial issue on both theoretical and methodological grounds (see, e.g., Kröll et al.^5^ for discussion). In typical recognition tests of contextual cueing, repeated displays are presented alongside newly generated arrays in randomized order. Participants are instructed to discriminate a given display as repeated versus non-repeated (i.e., foil display). These yes/no recognition tests typically fail to find statistical evidence of explicit recognition of repeatedly encountered displays (see, e.g., Goujon et al.^9^ for review), though increasing the test power has been shown to be sufficient to yield above-chance recognition performance^10^. Furthermore, a meta-analysis by Vadillo et al.^4^ showed that even though single studies may not show explicit recognition of repeated search displays, the overall evidence points to above-chance recognition performance, again indicating that individual tests are hampered by lack of statistical power. Accordingly, Shanks and collaborators^4,11^ concluded that contextual cueing is based on a single, explicit memory system. However, this view was challenged recently by Colagiuri and Livesey’s^6^ investigation of contextual cueing in very large samples. They found no relationship between cueing and awareness, leading them to conclude that contextual cueing is based on non-conscious learning and that cueing and recognition are driven by two independent memory systems. Arguably, though, all these studies, and their discrepant conclusions, suffer from one shortcoming: the absence of an active manipulation of the type, and with it: diagnostic accuracy, of the explicit test. While previous work examined for explicit recognition of repeated displays using almost exclusively yes/no recognition tasks, the present experiment examined for explicit knowledge of using a different—target-quadrant generation—task^10–12^, hypothesized to provide a more sensitive measure than the yes/no task^11^. Our approach was to compare the accuracy of the generation test with that of the yes/no test on a variety of performance measures derived from different memory models that assume in-/dependence of implicit and explicit processing.

### Rationale of the present study

The current study was designed to investigate how the memory architecture underlying scene-based spatial learning in visual-search and explicit-recognition tasks is to be characterized. Accordingly, the experiment consisted of two parts: First, participants performed a visual search task in which repeated display layouts were shown together with non-repeated (‘baseline’) displays. Thereafter, explicit-memory tests were administered to assess participants’ conscious awareness of the repeated displays (cf. Chun and Jiang^2^). Two types of explicit tests were used: yes/no recognition versus target-quadrant generation. The yes/no recognition task required participants to judge each repeated (vs. new) display as a repeated versus baseline (i.e., ‘foil’) display. In the target-quadrant generation task, participants again encountered repeated (vs. new) displays. However, this time the target (in both repeated and baseline displays) was replaced by an additional distractor item. The task was to indicate—or ‘generate’—the quadrant of the substituted target item, by pressing the response key associated with the respective quadrant. Successful performance of the target-quadrant generation task would strongly rely on memory of the positioning of the target relative to the distractors in its vicinity. A total of eight repeated displays, each with a unique spatial layout, were presented together with non-repeated displays during both the search and explicit memory tasks. We expected memory effects to manifest in both tasks to be performed: during *search*, activation of acquired context memories by repeated configurations should yield shorter reaction times (RTs) as compared to baseline displays; in the *explicit-memory tasks*, memory access should improve ‘recognition’ of repeated displays. Our particular focus was on how spatial memory for repeated displays expressed in the search task would relate to measures of memory obtained in the explicit tasks.

### Model predictions

#### One memory system

The first account assumes the existence of a single memory system underlying both contextual cueing of visual search and recognition of repeated displays^4,5,10,11^. On this view, the effects of acquired contextual cues should be evident in both the search task and the memory test, with performance being based on the same explicit memory system (provided the experiment is adequately powered and the measures have good reliability^4^). That is, contextual facilitation should manifest in both the visual search and explicit-memory tasks, with the dependent measures in the two tasks exhibiting a significant correlation.

#### Two memory systems

The second model assumes that contextual cueing is supported by two independent memory systems^2,6,7,9^: one is implicit, driving contextual cueing in the visual-search task; and the other is explicit, facilitating conscious retrieval of context cues in the explicit-memory task. Accordingly, there should be no systematic correlation between measures of contextual facilitation obtained in the search and memory tasks.

#### Display properties and contextual cueing

While previous studies agree that, in a given contextual-cueing experiment, only some 30% of repeated displays are actually learnt and thus come to produce a RT benefit in the search task^11,13^, it is less clear which perceptual factors, or visual display properties, modulate the learning of individual repeated displays. Olson and Chun^14^ found visual hemifield differences in contextual cueing: the cueing effect was greater for displays with targets appearing in the right visual field. But the targets’ horizontal display location may not be the only factor impacting the learning of repeated target-distractor arrays. For instance, in a systematic analysis of contextual cueing as a function of the placement of the target in the visual display, Zinchenko et al.^15^ found that targets located nearer to the display center yielded stronger contextual-cueing effects compared to targets positioned further away.

Moreover, subjective display properties, such as observers’ judgment of the ‘goodness’ of the visual display arrangement, are positively related to contextual learning^16^. Confirmatory evidence for the role of subjective factors in contextual cueing comes from pilot work we conducted in the course of the present study (see pilot study 1 below). The main finding was that people verbally described seemingly random spatial patterns—which were later on, in the main experiment (performed by different observers), utilized as repeated layouts in the search and explicit recognition tasks—according to criteria such as grouping (i.e., whether individual items formed larger chunks or groups of items), regularity (whether a spatial pattern represented a nonaccidental [versus random] arrangement of individual elements), or symbolism (whether a spatial pattern signified a real-world conceptual structure, e.g., stars, butterflies, letters, etc.).

Thus, individual search displays can be ‘described’ in terms of both *objective* display characteristics (relating, e.g., to the horizontal position of the target) and *subjective* characteristics (relating, e.g., to the degree of experienced inter-element grouping), where both these ‘surface’ properties may influence contextual learning of these displays^14,16^.

Given this, it should be possible to estimate contextual cueing in the search and the explicit-memory tasks (by comparing the respective performance measures between repeated and non-repeated displays; = contextual-facilitation effect) and then (try to) predict the facilitation on the basis of a regression-based classification approach, with objective and subjective display characteristics serving as predictors. Arguably, such a regression-based approach would provide an extra angle to understanding how the memory system/s underlying context effects in search and explicit memory tasks is/are to be characterized. If contextual cueing is supported by a single, explicit, memory system, then the very same predictors should “load” on different measures of the contextual-facilitation effect obtained in different—the visual search and explicit memory—tasks.

In summary, analyzing contextual cueing at the level of individual search arrays, while at the same time quantifying these displays in terms of (objective, subjective) spatial layout information, opens up new possibilities for the test of the above models on the coupling of contextual cueing in visual search and explicit memory tasks.

## Methods

### Participants and setup

60 participants took part in the experiment (24 male; mean age: 27.4 years). All reported normal or corrected-to-normal vision and were naïve as to the purpose of the study. The study was approved by the Ethics Committee of the LMU Psychology Department in accordance with the Declaration of Helsinki, and all procedures were carried out in accordance with the declaration’s guidelines and regulations. Participants provided written informed consent prior to the experiment and received either course credit or payment of 9 Euro (~ 10 USD).

### Stimuli and procedure

The experiment was conducted on a Windows PC with purpose-written experimental control software (in C++). Participants sat in a dimly lit laboratory in front of a 19-inch CRT monitor [AOC, Amsterdam, NL] with a resolution of 1024 × 768 pixels (refresh rate: 85 Hz) at a viewing distance of 57 cm. Search displays contained 12 black items (1.00 cd/m^2^; size: 0.55° × 0.55°) presented on a white screen background (25.40 cd/m^2^). There were 11 L-shaped distractor items which were rotated by angles of 0° (┗), 90° (┏), 180° (┓), or 270° (┛), and one T-shaped target item, which was rotated by either 90° (┫) or 270° (┣). Items were placed on four imaginary concentric circles around the center of the display, with radii of 2.19°, 4.10°, 6.60°, and 8.80°, respectively. Items were positioned such that there were at least three items in each display quadrant. Target locations (in repeated and non-repeated displays) were pseudo-randomized such that targets appeared equally often on ring 2 versus ring 3, in the left versus right display half, and in the upper versus lower display half.

#### Search task

Each trial started with the presentation of a black fixation cross (1.00 cd/m^2^; 0.5° × 0.5°) in the center of the computer monitor for a variable time interval of 700–1500 ms. After a blank interval of 20 ms, the search displays appeared. Each display was presented until observers made a speeded response (or a maximum display duration of 5000 ms had elapsed) by pressing the right or left arrow key of the computer keyboard depending on the corresponding orientation of the target (90° and 270° orientation, respectively). Correct responses were followed by a blank screen of 500 ms. In case of response errors, a warning message (“Fehler”—German word for error) appeared on the screen for 1000 ms, which was followed by a another (blank) interval of 1000 ms until the next trial began. The visual search task comprised 576 trials, divided into 36 blocks of 16 trials each. In each block, there were eight repeated displays together with eight non-repeated (baseline) displays. The repeated displays were identical for all participants and generated at the beginning of the pilot studies (see below). Non-repeated displays were generated anew on each trial. Targets appeared at a fixed set of 16 locations throughout the entire experiment: 8 locations were used for repeated displays, and 8 (other) locations for non-repeated displays. With the latter, we controlled for target location repetition effects across the two types of displays. Thus, any beneficial effects arising from repeated displays could only be attributed to the effects of repeated distractor-target contexts rather than absolute target positions in these displays.

#### Yes/no recognition task

The yes/no task had 128 trials (i.e., 8 blocks × 16 trials) and was performed in close succession to the search task. Each block had eight repeated displays from the search task and eight newly composed (foil) displays, presented in random order. Note that the new displays were also repeated across consecutive blocks of the yes/no task. This manipulation allowed us to equate stimulus repetitions across the two types of (repeated, non-repeated) display, so that learning within the yes/no task could itself not improve recognition performance. Each display contained 11 letter “L” distractors (presented in one of four orientations: 0°, 90°, 180°, 270°) and one (90° vs. 270°) letter “T” target. Participants were asked to indicate whether or not they had seen a given display during the previous search task by pressing the corresponding (Y or N) key on the computer keyboard. Recognition responses were non-speeded and no error feedback was provided.

#### Target-quadrant generation task

The target-quadrant generation task followed the search task and again consisted of 128 trials (i.e., 8 blocks × 16 trials). Each block had 8 repeated displays (from the search task) and 8 newly generated (foil) displays. The foil displays were also repeated throughout the generation task, again in an attempt to control learning within the test itself. Further, in foil displays, the distractors substituting the target were also shown at a fixed set of 8 locations (previously used in the search task).

Participants were informed that they would again see the repeated displays from the previous search task (together with newly composed foil displays). However, this time the “T” target letter was replaced by a (randomly oriented) “L” distractor item. In response to the test display, participants’ task was to indicate the quadrant in which (in the previous search task) the target “T” had been presented (now substituted by an “L” distractor item). Responses were given on the numeric keypad on the right-hand side of the computer keyboard, using spatially corresponding keys: “7” key for targets located in the top left display quadrant; “9” key for the top right quadrant; and the “1” and “3” keys for targets at the bottom left and right quadrants, respectively. Observers’ responses were non-speeded. No error feedback was given.

#### Pilot study 1: collecting observers’ terms for spatial patterns

**Figure 1:**
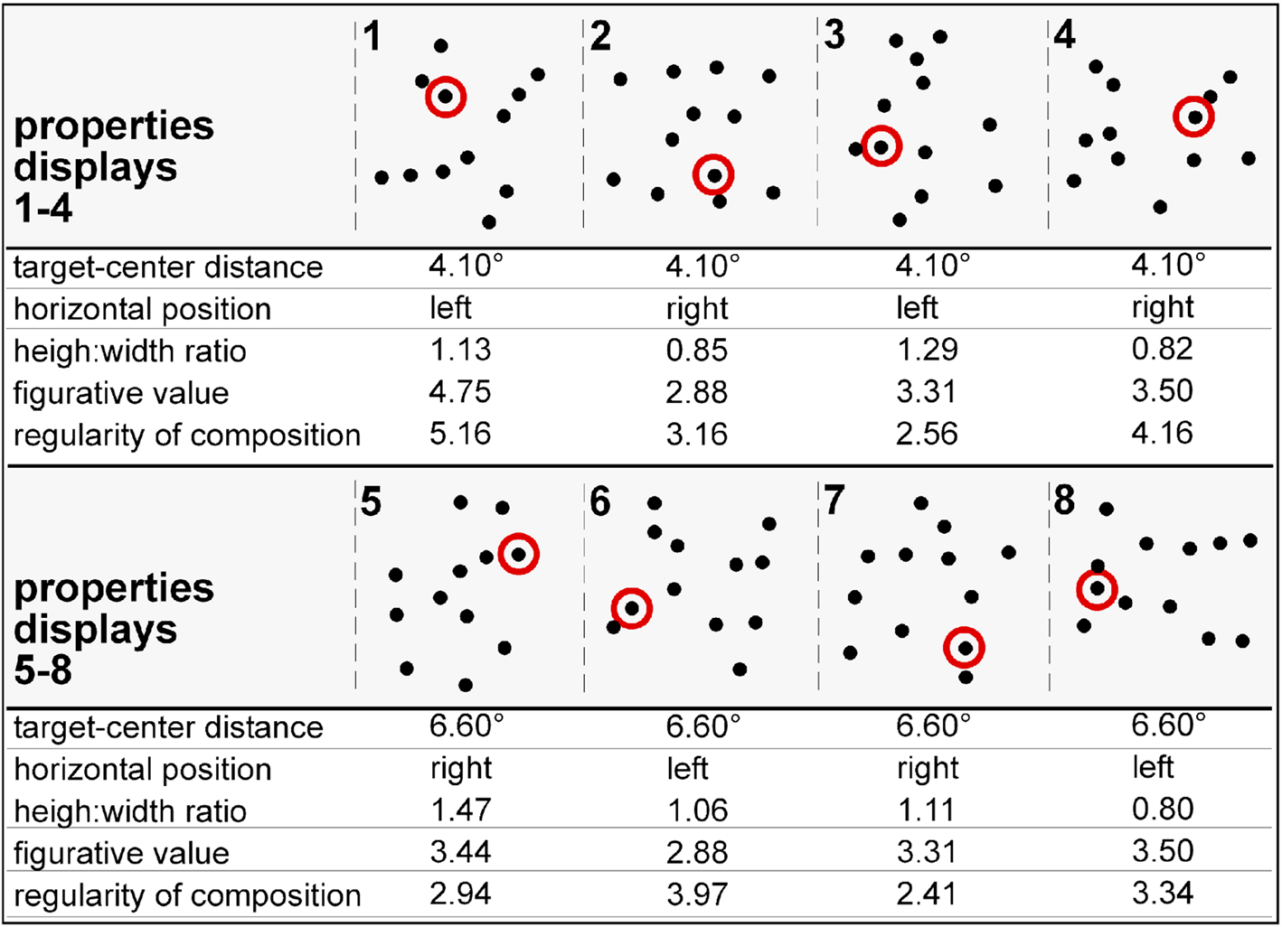
Spatial dot patterns evaluated in the pilot studies (for the purpose of illustration, the patterns are arbitrarily labelled as 1–8). In pilot study 1, participants had to give terms that they would use to describe the eight patterns. These terms were then classified into three larger categories (figurative value, inter-element grouping, regularity of pattern composition). In pilot study 2, participants rated individual patterns in terms of their figurative value (scale from 6 [high) to 1 [low]), inter-element grouping (scale from 6 [high] to 1 [low]), and regularity of composition (scale from 6 [systematic] to 1 [random]). The individual patterns were then introduced as repeated displays in the search and explicit memory tasks in the main experiment. This was achieved by replacing the 12 dots with 11 distractor ‘L’ letters plus 1 target ‘T’ letter. For each pattern, the dot later becoming the target is marked by a red circle (not shown in the actual search and explicit memory tasks). Further, below each pattern, information is provided about the distance of the ‘target’ dot from the display center (measured in degrees of visual angle), its (left vs. right) horizontal position, and the height-to-width ratio of a given pattern (values > 1 indicate that the display is spread in vertical direction). The figure also gives participants’ mean ratings for the two subjective ratings of figurative value and regularity of spatial composition. Figure drawn in Microsoft Powerpoint for Mac (version 16.16.05; URL: https://office.microsoft.com/powerpoint).

13 participants (male: 4; mean age: 26.1 years; all native German speakers) took part in pilot study 1, the aim of which was to investigate which language terms people would use when describing patterns of dot stimuli. These patterns were identical to the eight repeated arrays used in the search/explicit memory tasks. Thus, the very same arrays were used to assess participants’ subjective experience and contextual memory of these configurations (see Fig. 1). All participants were psychology undergraduate students and took part in the study during a seminar at LMU Munich. The 8 spatial patterns were printed on a single sheet of (A4) paper in landscape orientation, arranged on an invisible grid of 2 columns × 4 rows. Participants were told that “In this study, we are interested in how you would use to describe these spatial patterns using terms from daily language”. Participants were asked to write down as many terms as came to mind, taking into consideration the entire set of (8) arrangements, rather than only describing individual patterns. The task took participants about 5 min to complete. They wrote down their descriptions on a separate (blank) sheet of paper, which was collected afterwards by the course lecturer (TG).

Participants produced a total of 103 descriptions (i.e., single words, short phrases, or whole sentences), which were then classified into broader categories by two experts involved in research on contextual cueing. The resulting classifications were compared and possible discrepancies were discussed with a third expert (TG) to arrive at a single solution. 102 of the original 103 descriptions (= 99%) could be classified into 3 larger categories: (1) *Figurative value*—indexing the capability of dot patterns to signify meaningful, real-world entities; example descriptions include “teddy bear”, “tree”, “star” constellation, or “soccer tactics”. (2) *Inter-element grouping*—indicating whether speakers ‘see’ individual dots as aggregated in larger chunk or group/s of items; example descriptions are “many points but no overall gestalt”, “connection”, “bisected”, “need to encircle the dots in subsets”. (3) *Regularity*—signifying rules or principles that may have led to the construction of the patterns; example descriptions are “the dot patterns seem to be chaotic through and through”, “deliberate and not accidental arrangement”, “orderly chaos”, or “confusion”.

#### Pilot study 2: collecting observers’ subjective experiences of spatial patterns

Another sample of 16 (different) psychology students (2 males, mean age: 23.1 years) received the 8 spatial patterns and had to rate them according to the main categories derived from pilot study 1. This was done by presenting them with 3 sheets of papers (in a fixed, sequential order), each containing the 8 patterns (presented in the same 4 × 2 grid as in the pilot study 1). On sheet 1, participants were asked to rate the *figurative value* of each individual pattern on a scale from 6 (high) to 1 (low). Sheet 2 required them to rate the *inter-element grouping* of each pattern from 6 (high) to 1 (low). On sheet 3, they had to judge the *regularity* of the composition the individual patterns using a scale from 6 (systematic) to 1 (random). Prior and during each rating, participants were presented (via video projection) with relevant category-specific example terms/descriptions obtained from pilot study 1 to ensure that they understood the concepts behind each scale. For each pattern and rating, participants were given a fixed time of 10 s (controlled by a stop clock) before being prompted (verbally) to move on to the next pattern. There was a 1-min break between the three subjective ratings.

The data were then further evaluated by a (confirmatory) factor analysis, with participants ratings as rows and expert categories as columns. To quantitatively determine whether the experts’ category solutions were acceptable, we compared a full factor model (assuming that the three expert scales represent separable latent dimensions) with a model that constrained the number of underlying variables. The latter was based on an a-priori (correlation) analysis of observers’ subjective rating data, which revealed the ratings of inter-element grouping and pattern regularity to have a significant positive relationship, r = 0.27, *p* < 0.01 (all other p’s/BF10′s < 0.13/0.61). Given this finding, we compared the full—3-factor—model with a constrained—2-factor—model, the latter fitting (1) the ratings of *symbolism* and (2) the averaged ratings of inter-element grouping/pattern regularity (henceforth referred to as the single factor *regularity of spatial composition*). Model comparisons were realized using the Akaike information criterion (AIC)^17^, with lower AIC values signifying better model fit. The sub-model with factors for *symbolism* and (combined) *regularity of spatial composition* outperformed the full model: the AIC value was lower for the sub-model than for the full model, 892 versus 1413. Thus, according to (confirmatory) factor analysis, the two dimensions of symbolism and regularity of spatial composition suffice to describe participants’ rating data. For this reason, we adopted the two-dimensional solution for describing participants’ experiences of spatial patterns and the impact of these subjective factors on contextual cueing and explicit-memory performance. Participant ratings are summarized in Fig. 1.

#### Design and analysis

We recruited 60 new observers (who had not participated in any pilot study) for the main experiment (see above), consisting of one search and one explicit-memory task: either yes/no recognition or the target-quadrant generation; each 30 participants were randomly assigned to perform one or the other of the latter tasks. Prior to each task, participants received written instructions informing them about the respective task to be performed. For the explicit-memory (but not the search) tasks, the instruction included information about the fact that 50% of the display arrangements presented for recognition had previously been encountered repeatedly during the search task. All observers started the experiment with the visual search task and then went on to perform either the yes/no recognition or the target-quadrant generation task. The main experiment took some 50 min to complete. The experimental variables were Context (repeated display, non-repeated display; within-subject variable), Epoch (1–6; one epoch encompasses six blocks of trials in order to obtain a reasonably stable estimate of contextual-cueing effects; cf. Chun and Jiang^2^), and Explicit Memory Task (yes/no recognition, target-quadrant generation; between-subject variable). For the yes/no task, we compared observers’ hit responses (correct judgement of repeated display as “repeated”) with their false-alarm responses (incorrect judgement of non-repeated display as “repeated”). For the target-quadrant generation task, explicit knowledge about repeated search arrays was assessed by comparing hit rates (correct detection of the quadrant of the substituted target) between repeated and non-repeated displays. Note that for both repeated and non-repeated displays, targets were substituted by distractors, with the distractors shown at the same fixed sets of 2 × 8 target locations previously used in the search task.

#### Single-display analysis

Chun and Jiang^12^ have shown that the decrease in RTs across trial blocks/epochs attributable to contextual cueing can be described almost perfectly by an exponential (i.e., power) function, where the critical parameter indicative of learning is provided by the (negative) exponent of this function. In the subsequent *single-display analysis*, we determined learnt displays by applying a two-parameter power function of the form $RT=i\times {x}^{s}$ (cf. Brooks et al.^18^) to RTs for each individual repeated display (and non-repeated, baseline, displays). Parameter *i* corresponds to the intercept of the RT × epoch function, and parameter *s* to the (negative) slope of the function; *x* represents the search epoch (1–6). After quantifying the slopes for each individual display (and observer), we calculated the difference in slopes between a given repeated display and the mean slope obtained from all non-repeated displays. This was based on the observation that in visual search tasks, RTs typically decrease with increasing epoch number for both repeated and non-repeated displays (an effect attributable to perceptual or skill learning^19^), while this practice-dependent RT facilitation is typically greater for repeated displays (= contextual-cueing effect). When subtracting the (negative) exponents of the function relating RTs to epoch number between repeated and non-repeated displays, negative difference values would thus indicate ‘true’ context learning (without contributions from non-configural, skill learning).

Contextual learning of individual displays was also assessed in the explicit tasks. Specifically, for the yes/no task, contextual cueing was estimated by comparing the hit rate from each repeated display (correct recognition of repeated display as ‘repeated’) with the mean false-alarm rate obtained from all non-repeated displays (erroneous recognition of non-repeated displays as ‘repeated’). Explicit performance in the target-quadrant generation task was evaluated on the basis of the comparison between hit rates to individual repeated displays (correct judgement of the [substituted] target quadrant) with the mean hit rate obtained from non-repeated displays.

## Results

Data analysis was performed using R^20^. In case of non-significant effects, Bayes analyses were performed in order to quantify the evidence for the null hypothesis. Bayes Factors were calculated using the package BayesFactor^21^. BF10 values lower than 1 provide weak evidence and values lower than 0.30 substantial evidence for the null hypothesis^22^. Error trials and trials with extreme RTs (outside 2.5 SDs from the individual mean) were discarded (1.62% and 2.55%, respectively). The first five trials in the search and explicit memory tasks served as practice trials (data not recorded).

### Search task

**Figure 2:**
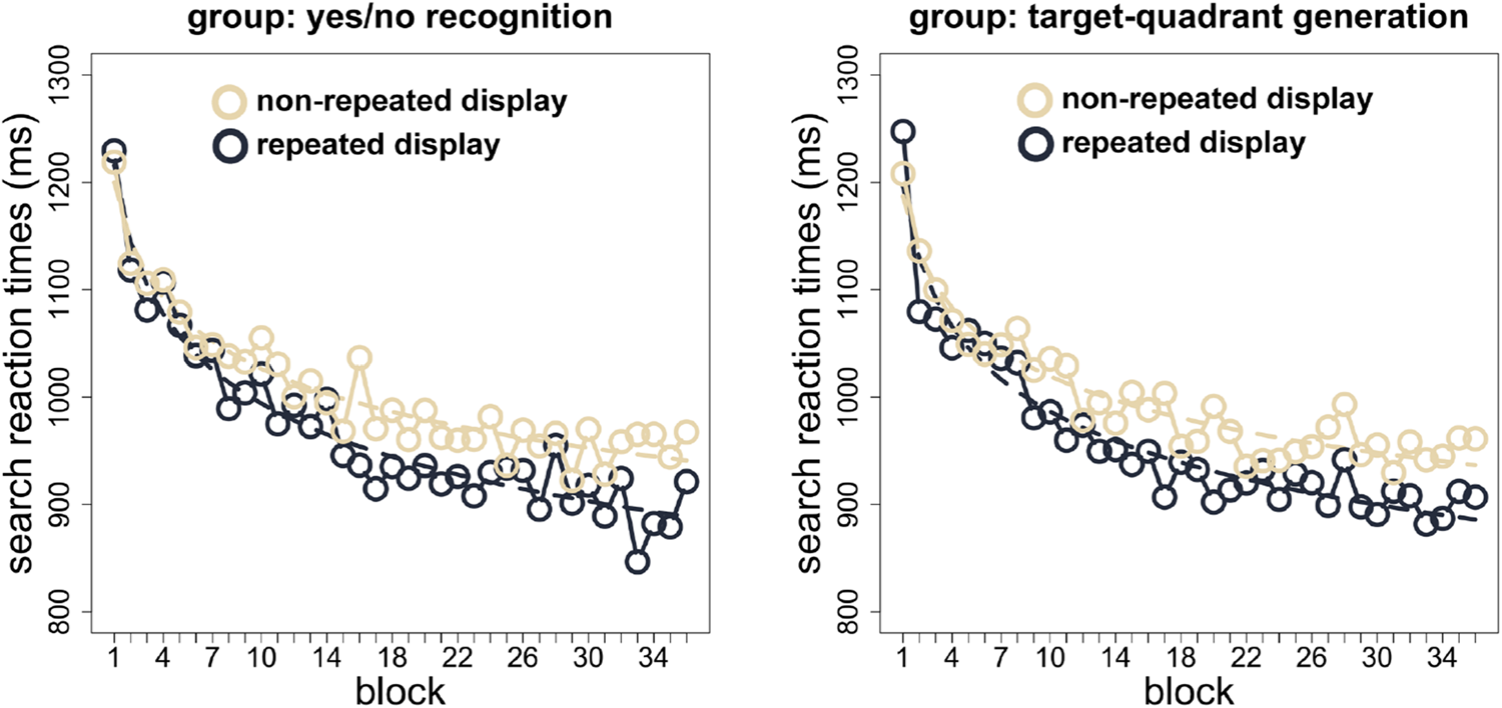
Search reaction times (in milliseconds) for repeated and non-repeated displays in each of the 36 experimental blocks (each block consisted of 8 trials with repeated and another 8 trials with non-repeated displays) for observers who later on performed the yes/no recognition or the target-quadrant generation task (left and right panels, respectively). The dashed lines represent two-parameter power-function fits to the data^18^ Figure drawn in R^20^.

Repeated displays elicited faster RTs than non-repeated displays, an effect that became more pronounced as the experiment progressed, F(5,290) = 3.78, *p* < 0.05 (significant context × epoch interaction). The magnitude of contextual cueing (RT non-repeated display minus RT repeated display) in the first half of the experiment (epochs 1–3) was 30 ms, which compares with an effect of 45 ms in the second half (epochs 4–6; 30 vs. 45 ms; one-tailed t(59) = 2.51, *p* < 0.01, Cohen’s d = 0.32, 95% CI [− 0.04, 0.68]). Importantly, contextual cueing effects were statistically indistinguishable between observers who later participated in the yes/no and, respectively, the generation task, F(5,290) = 1.40, *p* = 0.22, BF10 = 0.14 (non-significant context × epoch × group interaction): the mean contextual-cueing effect was 38 ms for observers who performed the yes/no task (repeated RT: 941 ms; non-repeated RT: 979 ms; one-tailed t(29) = 5.05, *p* < 0.01, Cohen’s d = 0.26, 95% CI [− 0.25, 0.78]), and 37 ms for observers who completed the generation task (repeated RT: 939; non-repeated RT: 976 ms; one-tailed t(29) = 5.21, *p* < 0.01, Cohen’s d = 0.29, 95% CI [− 0.23, 0.80]). Thus, it is unlikely that differences in the ability of the two memory tests to reveal evidence for explicit cueing is attributable to differences in the overall strength, or magnitude, of contextual facilitation achieved in the search task. See also Fig. 2, which depicts how reaction times to repeated and non-repeated displays developed over time, for the two groups.

### Yes/no recognition

In the yes/no recognition test, conducted after the search task, the hit rate (correct recognition of repeated displays as repeated) was higher than the false alarm rate (erroneous recognition of non-repeated display as repeated), 49.9% versus 46.2%, one-tailed t(29) = 1.80, *p* = 0.08, Cohen’s d = 0.28, 95% CI [− 0.24, 0.79], though there was only inconclusive evidence for the null hypothesis, BF10 = 0.82.

### Target-quadrant generation

For observers who performed the target-quadrant generation task, the ability to consciously recollect a repeated display was assessed by comparing their hit rates between repeated and non-repeated displays. A hit means that observers correctly indicated the quadrant of the substituted target. Since observers could solve this task only by chance in non-repeated displays (i.e., 25% correct), chance performance in the memory test is indicated by comparable performance between the repeated and non-repeated conditions. However, a one-tailed t-test confirmed the hit rate to be significantly higher for repeated than for non-repeated displays: 31.8 versus 27.7%, t(29) = 3.01, *p* < 0.01, Cohen’s d = 0.69, 95% CI [0.16, 1.23].

### Cross-test comparison

Although the effect size was numerically greater in the generation versus the recognition test (0.28 vs. 0.69), the difference was not significant (two-sample z-test: z = − 0.60, *p* = 0.39). This suggests that, in principle, both tests have power to reveal evidence of explicit memory for repeated displays. Since the recognition and generation tasks differed with regard to baseline—chance—performance (50 vs. 25%), in order to ensure comparability between the two types of test, we normalized test performance by relating individual participants’ scores to chance performance. That is, for the recognition task, we first computed a difference score (hit rate for repeated displays minus false-alarm rate for non-repeated displays) for each participant and then divided this score by her/his false-alarm rate (= baseline performance). For the generation task, scores were obtained by calculating the difference between individual participants’ hit rate for repeated displays and their hit rate for non-repeated displays and dividing this score by their hit rate for (baseline) non-repeated displays. The normalized effect scores were 0.18 and 0.09 for the generation and yes/no tasks, respectively; that is, compared to the yes/no task, the generation was able to detect twice as many responses as being driven, or ‘facilitated’, by explicit context memory. Again, however, the difference was not significant (z = 0.58, *p* = 0.27).

Interestingly also, the two explicit measures of contextual facilitation were unaffected by test precision: mean split-half reliability estimated by the Spearman–Brown formula was 0.61, and this measure was near-equivalent for recognition and generation, r = 0.61 and r = 0.62, respectively (z = − 0.06, *p* = 0.95). Reliability coefficients were calculated by dividing the (128) explicit-memory trials into four epochs (of 32 trials each) so as to obtain reasonably stable estimates of conscious display recognition in each epoch (consisting of 2 × 8 old and 2 × 8 new displays). This yielded four recognition scores per participant (yes/no task: **|**hit rate for repeated displays minus false alarm rate for non-repeated displays**|**; generation task: **|**hit rate for repeated displays minus hit rate for non-repeated displays**|** for the generation task), which were then divided into two halves using an odd–even split and correlations were computed.

### Relation between measures of contextual cueing obtained in the search and explicit memory tasks

**Figure 3:**
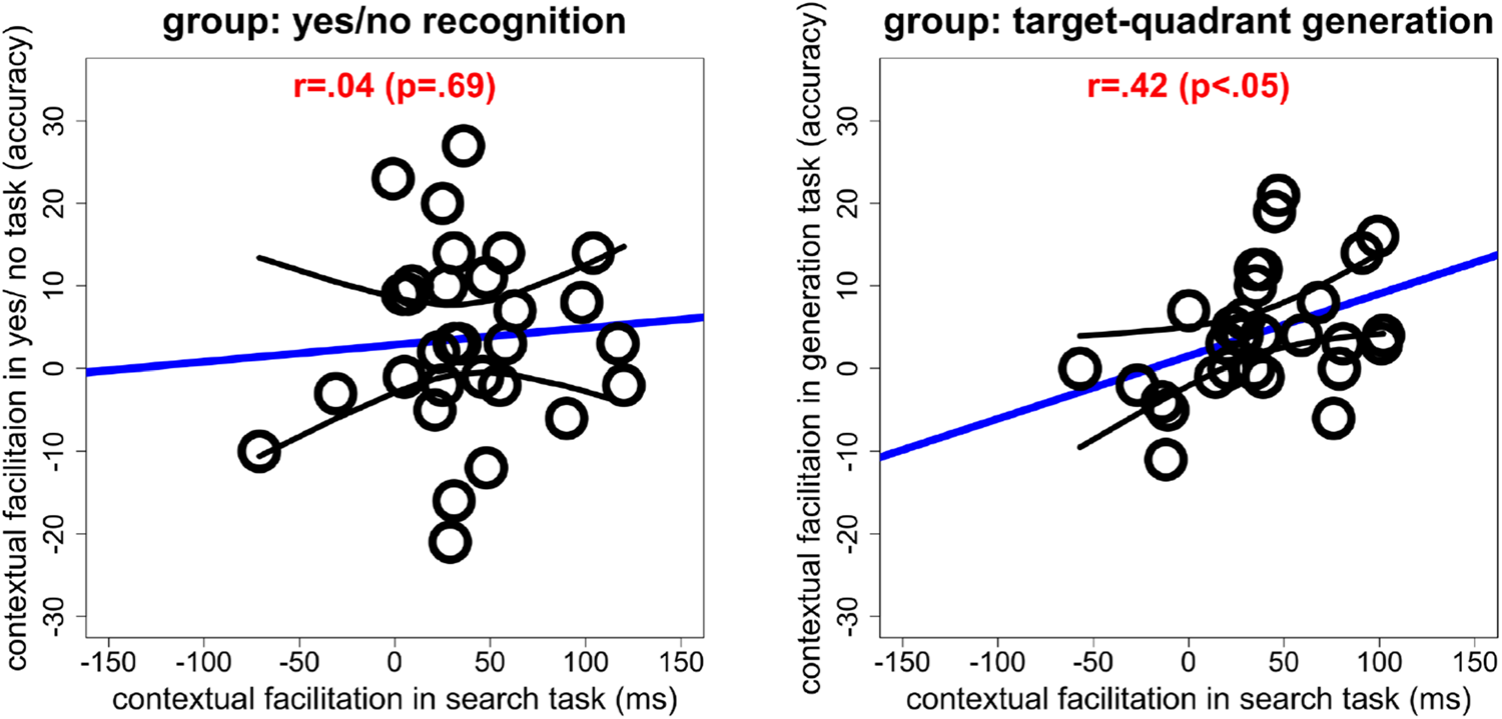
Correlation between contextual facilitation in the visual search and the yes/no recognition tasks (left panel) and between contextual facilitation in the visual search and target-quadrant generation tasks (right panel). Contextual facilitation is assessed by comparing the respective performance measures between repeated and non-repeated displays. The black lines denote the 95% confidence interval. Figure drawn in R^20^.

Another strong prediction following from single-memory accounts is that different measures of contextual memory should exhibit a significant positive correlation. Indeed, as can be seen from Fig. 3, individual participants’ contextual-facilitation effects correlated significantly with their generation performance: r = 0.42, *p* < 0.05, 95% CI [0.05, 0.67]; that is, facilitation in the search task was predictive of recognition performance in the memory test. No such effect was found for observers who performed the yes/no recognition task: the correlation between their contextual-facilitation scores in the search and recognition tasks was non-significant: r = 0.04, *p* = 0.69, 95% CI [− 0.41, 0.31], BF10 = 0.24. Of note, though, comparing the two correlations by means of a z-test failed to reveal the difference to be significant: z = − 1.35, *p* = 0.17. Nevertheless, the correlation analysis tends to reinforce the findings, and interpretations, of the (signal-detection) accuracy measures presented above, namely, that the generation test has higher diagnostic accuracy than the recognition test in revealing evidence of explicit cueing.

### Predicting contextual cueing from objective and subjective display parameters

Exploring the impact of the objective display parameters (target-center distance, horizontal target position, height:width display ratio) and the subjective parameters (figurative value, regularity of composition) on contextual cueing during the *search task* yielded significant (linear) regression models: F(5,224) = 4.06, *p* < 0.01 for the yes/no group, and F(6,191) = 5.08, *p* < 0.01 for the target-quadrant generation group. As summarized in Table 1, both regression models yielded significant coefficients for target-center distance (indicative of better contextual learning for more ‘central’, relative to more ‘peripheral’, targets) and horizontal target position (indicative of better learning of targets located in the right, relative to the left, display half). For all other (mainly subjective) display parameters, the *p* values were quite high (> 0.18), that is, they bore no systematic relationship with the contextual-cueing effect obtained in the search task. Significant regression models were also found for *explicit-memory* performance: yes/no recognition, F(6,229) = 2.47, *p* < 0.05, target-quadrant generation, F(5,227) = 7.13, *p* < 0.01, though these model fits were qualitatively different from those in the search task. In more detail, explicit memory performance correlated significantly with the subjective display parameter of symbolic value (patterns with higher real-world ‘significance’ outperformed those of lower figurative value), in both the yes/no recognition task and the target-quadrant generation tasks (see Table 1). Interestingly, for the latter, there was also a systematic relationship between the distance of the target from the display center and explicit generation performance (quadrants with more central targets were recalled better than quadrants with more peripheral targets). In other words, the same—objective—display parameter predicted context-based performance gains in the visual-search and target-quadrant generation tasks. Moreover, for the generation task, there was also an effect of a given display’s height-to-width ratio (with better explicit performance for patterns that were spread more in vertical than in horizontal direction). For the yes/no task, by contrast, there was a systematic relationship between explicit performance and regularity of spatial composition (with regular, rule-based patterns having a higher likelihood to be recognized), besides that between memory performance and figurative value.

**Table 1:** Estimated regression coefficients and associated t statistics from analyses of the relationship between (objective, subjective) display features and contextual cueing in the search and explicit memory tasks.

	Contextual cueing in search task		Contextual cueing in yes/no recognition task	
	Estimate	t statistic	Estimate	t statistic
**Group: yes/no recognition**				
Intercept	− .0434	− .62	25.2160	1.34
Target-center distance	.0018	2.66**	− .3337	− 1.79
Horizontal position	− .0390	− 2.87**	− 3.7577	− 1.03
Height:width ratio	− .0124	− .34	14.4918	1.62
Figurative value	.0053	.31	− 12.4120	− 2.72**
Regularity of composition	− .0150	− .133	6.8483	2.26*

	Contextual cueing in search task		Contextual cueing in target-quadrant generation task	
	Estimate	t statistic	Estimate	t statistic
**Group: target-quadrant generation**				
Intercept	− .0716	− 1.50	9.6884	.59
Target-center distance	.0014	3.16**	− .3611	− 2.25*
Horizontal position	− .0262	− 2.83**	1.8731	.59
Height:width ratio	.0004	.21	38.6552	4.97***
Figurative value	.0039	− .35	− 11.7372	− 3.03**
Regularity of composition	− .0005	− .07	2.7986	1.08

For the search task, contextual cueing arising from individual search arrays was assessed by calculating the (negative) slope of the function relating RTs to epoch number and comparing the slopes between each repeated display and the mean slope for non-repeated displays. RTs were fitted by two-parameter power-functions. For the yes–no recognition task, contextual cueing was estimated by contrasting the hit rate for each repeated display (correct recognition of repeated display as ‘repeated’) with the mean false-alarm rate obtained from all non-repeated displays (erroneous recognition of non-repeated displays as ‘repeated’). Explicit performance in the target-quadrant generation task was evaluated on the basis of the comparison between hit rates to individual repeated displays (correct judgement of the [substituted] target quadrant) with the mean hit rate obtained from non-repeated displays.

Asterisks represent significance levels: ***.0001; **.001; *.01.

An alternative way to look at the data is to compare multiple-regression models with different underlying factors and examine which model/s best fit contextual facilitation in the search and explicit-memory tasks. In doing so, we also tested another class of—mixed—regression models, in addition to ‘standard’ models, allowing the models’ intercept to vary across different repeated displays (and participants). This mixed-model approach appears justified given the same set of (eight) repeated displays was used in the current study—thus, measures of contextual facilitation may be bound to these displays (and participants). Effectively, in the mixed-effect models, we treated individual repeated displays and participants as random factors; the fixed factors were our objective and subjective displays parameters.

**Figure 4:**
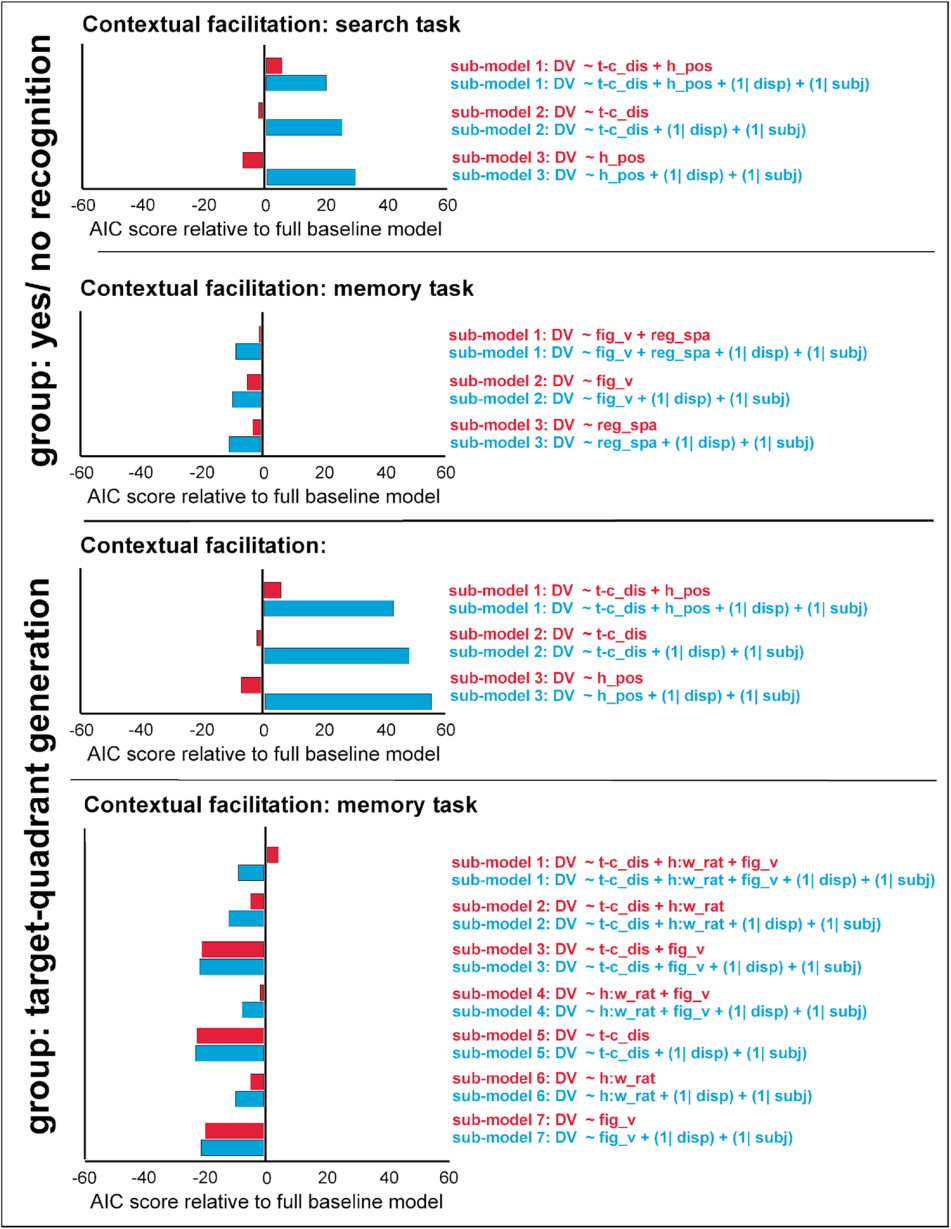
AIC model comparison. Akaike-information-criterion (AIC) values are compared between several sub-models of the full baseline model assuming contextual facilitation in search and explicit-memory tasks to vary as a function of objective display parameters (target-center distance, horizontal position, height:width ratio) and subjective parameters (figurative value, regularity of composition). Each sub-model has only one, two, or three predictors compared with the full model. Comparisons are made for two types of regression models that consider only variation that is explained by the independent variables of interest versus variation that derives from the independent variables of interest plus random effects (red and blue colors, respectively). Positive (negative) AIC values represent better (poorer) fit of a given sub-model relative to the full baseline model. DV: dependent variable. t-c_dis: target-center distance. h_pos: horizontal target position. h:w_rat: height:width ratio. fig_v: figurative value. reg_spa: regularity of spatial composition. disp: individual repeated display. subj: individual participant. Figure drawn in Microsoft Powerpoint for Mac (version 16.16.05; URL: https://office.microsoft.com/powerpoint).

Figure 4 depicts the relative AIC values for a set of sub-models compared to two baseline models, each assuming a relationship between the full set of—objective and subjective—predictor variables and contextual facilitation in the search and explicit-memory tasks. Baseline model 1 was a standard regression model including only fixed variables (of target-center distance, horizontal position, height:width ratio, figurative value, regularity of composition), while baseline model 2 was a mixed-effects model with both our set of objective/subjective parameters as fixed factors and repeated display and participant as random factors. Each sub-model, by contrast, involved only a reduced set of factors relative to the baseline models. In order to restrict sub-model space, we considered only predictors with significant regression coefficients obtained from the analysis of the full models (see Table 1). The AIC prioritizes models with fewer parameters, with smaller AIC values representing better model fit. Note, though, that since we present relative AIC scores [i.e., baseline model AIC minus sub-model AIC] in Fig. 4, positive values represent better sub-model fit.

For contextual facilitation in the *search task*, a model comparison demonstrated that sub-models with (only) target-to-center distance and horizontal position outperformed the full baseline model. This effect was seen with both (standard, mixed) regression baseline models. Concerning performance in the *explicit-memory tasks*, almost all sub-models had higher AIC values than the full baseline models (meaning that the full baseline model outperformed the sub-models), except the sub-model with target-center distance, height:width ratio, and figurative value, which performed better than the (standard-regression) baseline model.

In sum, analysis of the relationship between display properties and contextual cueing demonstrates that one set of—objective—display features is responsible for cueing in search tasks, while both sets of—objective and subjective—features influence performance in the explicit-memory tasks. For observers who performed the target-quadrant generation task, there was also a correspondence in the objective display feature of target-center distance, which could be used to predict contextual cueing in both the search and explicit-memory tasks. These findings were validated by a systematic regression-model comparison approach, which showed that sub-models involving the factor target-center distance outperformed the (full-factor) baseline models in the search and generation tasks.

## Discussion

### Summary of findings and interpretation

Contextual cueing refers to expedited visual search in repeated stimulus configurations. Despite this RT facilitation, observers seem to be poor in discerning the repeated patterns in (surprise) recognition tests performed immediately after the search task. This dissociation has been attributed to distinct unconscious and conscious memory systems driving search and recognition, respectively.

To distinguish between two- and one-system accounts of memory in contextual cueing, we used the novel approach of systematically testing the relationship between context-based RT facilitation in the search task and participants’ awareness of repeated search arrays in the memory task. Further, we compared and contrasted two statistically powerful explicit tests that hitherto have been used only in isolation: a yes/no recognition test and target-quadrant generation test. Furthermore, we evaluated the spatial properties of individual repeated arrays using objective and subjective descriptors and determined contextual-facilitation effects in search and explicit-memory tasks at the level of individual repeated arrays. This enabled us to test predictions about the relationship between measures of contextual facilitation obtained in the search and memory tasks, as well as examining for common versus separate regression coefficients for contextual- facilitation effects in these tasks.

Overall, our findings are in line with predictions derived from a one-system account. There was a search advantage for targets in repeated as compared to novel displays. This advantage was also observable in the target-quadrant generation (memory) task: observers were able to discriminate the quadrant of the substituted target in repeated displays well above chance level. Further, there was a significant correlation between measures of contextual facilitation in the search and explicit target-quadrant generation tasks. Additional linear-regression analysis revealed that this correlation was likely due to the search and generation tasks receiving support from common context representations relating to the placement of the target at more central versus more peripheral display locations. In contrast, signal-detection measures of explicit-memory for repeated displays and correlations between contextual facilitation in the search and memory tasks were less pronounced overall for the yes/no recognition test, even though this test was statistically as powerful as the quadrant-generation test (128 trials each)^4^. Accordingly, the generation test appears particularly apt in revealing explicit memory of repeated distractor-target arrangements in search tasks.

### Relations to other memory accounts

#### Transfer-appropriate processing

The differential power of the two tests to reveal evidence of explicit contextual facilitation may be best understood within the transfer-appropriate processing (TAP) framework^23–25^. According to this framework, the ‘success’ of the single memory system in facilitating conscious performance critically depends on the match of information required in the search and conscious-awareness tasks. The superiority of generation over recognition performance would then be attributable to greater similarity in terms of the processing requirements between the initial search and the subsequent memory task. In both tasks, observers had to localize the (substituted) target of their search; that is, both tasks rely on the same target-position-related mental knowledgebase.

In the yes/no recognition task, by contrast, observers encounter test displays in which all items, the target as well as the distractors, are visible and they have to simply indicate whether they believe they had seen a given display already during the previous search task. Given that a target is present in the (yes/no) test display, a recognition response may be given on the basis of other processes than those at work in the actual search and the target-quadrant generation task. For instance, observers may rely on their familiarity with the test displays. This is a kind of mere-exposure effect^26^, the idea being that repeated encounters of a stimulus on a later occasion induce subjective fluency, which suffices to judge this stimulus as old. Successful recognition may thus be supported by (fluency) processes that are different from the contextual representations facilitating reaction times and accuracy in the visual search (and the target-quadrant generation) task. Consistent with this, the regression analysis presented above indicated that successful recognition was influenced, or driven, by perceptual properties of the to-be-judged test displays, such as the figurative value of the test displays or the regularity of their composition, which were, however, qualitatively different from the objective parameters that drove performance in the search task.

Of note, the above account in terms of the TAP framework is only a post-hoc attempt to coherently explain the current findings. However, it makes predictions that can be tested in future research. Prior (eye-movement) studies have shown that, in the standard “T” versus “L” search task (which requires serial focal-attentional inspection of displays items), the contextual-cueing effect typically arises from the learning of only a few distractors within the local vicinity (e.g., the quadrant) of the target^27–29^. Assuming that the encoding of potentially search-guiding distractor-target relations occurs at the time point at which the target is detected^30^, it is not surprising that observers develop such more local context representations. Since we imposed no constraints on how our participants performed the search^15,31^, it is likely that they scanned the displays with a relatively narrow focus of attention, thus acquiring the local target context. The quadrant-generation task reinstated this local attentional set (scrutinizing the item arrangement within each quadrant to determine which one might have contained a target in the previous search), thus aiding the retrieval of the acquired, local context representations. However, it is also possible to make participants perform the search with a more distributed attentional set, which has been shown to foster the learning of the more global item arrangements^15^ see also Refs.^32–35^. It is conceivable that when contextual learning takes place with a more distributed attentional set (e.g., when participants are told not to move their eyes during the search^15^), global organizational properties of the search displays become more prominent in the acquired memory representation, so that the yes/no recognition test (where judgments were more reliant on the figurative value/regularity of the display arrangement) may outperform a target-quadrant generation test. Finding such a complementary pattern to that demonstrated in the present study would add support to a TAP-based account of conscious recognition performance.

#### Attention-to-memory model

Although the present results favor a one-system account of contextual cueing, they are consistent with variants of this account that (despite assuming only one, explicit-memory system) posit the involvement of functionally independent—un-/conscious—retrieval operations^36^. Applying this notion to the present visual-search and generation-test scenario: distractor-target representations will, at a first stage, be retrieved rapidly and automatically from long-term context memory, without conscious awareness—leading to contextual facilitation in the search task. This is followed by second, slower, retrieval stage at which memory contents become consciously accessible and can thus inform explicit responses. These accounts attribute an important function to focal attention in the transition of information from the first to the second stage, with attention enhancing (internal) memory signals in the same way as it enhances the processing of external stimuli. Given that performing the generation task requires attentional scrutiny of the various display quadrants, it is conceivable that memory representations associated with the local target quadrant (which are sufficient to facilitate search; see, e.g., Ref.^28^) are amplified by focal attention above some threshold for conscious report^10^. This would apply less to the yes/no recognition task, in which focal attentional engagement would be lower overall. A comparison of the response times in our two explicit-memory tasks provides tentative evidence for this idea: although participants were not instructed to respond as fast as possible, they took considerably longer to make their decisions in the generation, as compared to the recognition, task: 3,961 versus 2,319 ms, t(39.38), *p* < 0.01, Cohen’s d = 0.85, 95% CI [0.31, 1.39], consistent with increased attentional processing in the former task. Accordingly, focal-attentional scrutiny may boost (conscious) remembering.—We acknowledge, though, that the role of focal attention in mediating conscious retrieval of learnt distractor-target relations needs to be corroborated in purpose-designed research.

## Conclusion

Contextual cueing is an important predictive-coding mechanism, helping us to rapidly detect and respond to target objects in recurrent scenes^37^. Prior studies were inconclusive as to whether contextual learning involves an implicit or an explicit memory system, though the balance of evidence suggested a role of consciously accessible memory in contextual cueing^4,10^. Here, we show that (success in) revealing explicit knowledge of distractor-target relations (that renders contextual cueing in visual search) critically depends on the type of explicit-memory test employed: active ‘quadrant-generation’ tests are superior to passive ‘yes/no recognition’ tests. These results have implications for the memory architecture underlying context effects in visual search and explicit recognition. In particular, we suggest that both objective (i.e., physical) and subjective (i.e., conceptual) display properties come to be integrated in the memory for distractor-target relations during the search task. Because of this, significant correlations between different measures of the cueing effect obtained in search and explicit-memory tasks heavily rely on the specificity of the explicit test. The ‘target-quadrant generation’ and ‘yes/no recognition’ tasks involve different processing requirements and thus benefit from different types of—more spatial versus more conceptual—contextual information, where facilitation of search is predicted (i.e., facilitated) by the more spatial factors. Given this, future studies would be well advised to employ an explicit generation task that matches the typical, spatially focused processing requirements of the search task in order to investigate the memory representations underlying contextual cueing.

## Data Availability

Data and analysis scripts are available at osf.io/2ge9u.
